# RSA migration of total knee replacements: A systematic review and meta-analysis

**DOI:** 10.1080/17453674.2018.1443635

**Published:** 2018-03-06

**Authors:** Bart G Pijls, José W M Plevier, Rob G H H Nelissen

**Affiliations:** 1 Department of Orthopaedics, Leiden University Medical Center, Leiden; 2 Walaeus Library, Leiden University Medical Center, Leiden, The Netherlands

## Abstract

**Purpose:**

We performed a systematic review and meta-analyses to evaluate the early and long-term migration patterns of tibial components of TKR of all known RSA studies.

**Methods:**

Migration pattern was defined as at least 2 postoperative RSA follow-up moments. Maximal total point motion (MTPM) at 6 weeks, 3 months, 6 months, 1 year, 2 years, 5 years, and 10 years were considered.

**Results:**

The literature search yielded 1,167 hits of which 53 studies were included, comprising 111 study groups and 2,470 knees. The majority of the early migration occurred in the first 6 months postoperatively followed by a period of stability, i.e., no or very little migration. Cemented and uncemented tibial components had different migration patterns. For cemented tibial components there was no difference in migration between all-poly and metal-backed components, between mobile bearing and fixed bearing, between cruciate retaining and posterior stabilized. Furthermore, no difference existed between TKR measured with model-based RSA or marker-based RSA methods. For uncemented TKR there was some variation in migration with the highest migration for uncoated TKR.

**Interpretation:**

The results from this meta-analysis on RSA migration of TKR are in line with both the survival analyses results from joint registries of these TKRs as well as revision rates results from meta-analyses, thus providing further proof for the association between early migration and late revision for loosening. The pooled migration patterns can be used both as benchmarks and for defining migration thresholds for future evaluation of new TKR.

Worldwide, some several hundred thousand total knee replacements (TKR) are implanted every year. Aseptic loosening is the major reasonfor revision in the long term, with rates between 5% and 10% in large national registry databases (AOANJRR 2016, SKAR 2016). Early migration of tibial components of TKR, measured with radiostereographic analysis (RSA), has been associated with long-term risk of revision for aseptic loosening (Pijls et al. 2012a). Furthermore, continuous migration during the second postoperative year has been associated with early onset of loosening (Ryd et al. 1995). One may consider early migration of an orthopedic implant (maximal total point motion (MTPM) 1 year) to be associated with achieving fixation and therefore to provide information on the bone–prosthesis or bone–cement–prosthesis interface. The better the fixation, the lower the risk of aseptic loosening in the future. The worse the initial fixation, the higher the risk of aseptic loosening in the future. One may consider continuous migration (MTPM over 1–2 years) to be associated with early manifestation of pathology, i.e., early loosening (Ryd et al. 1995). If the continuous migration progresses it will eventually be radiographically visible (i.e., radiolucencies around the implant) and give rise to symptoms in the patient.

Although early migration and continuous migration are 2 completely different entities, they can be used in conjunction in a phased clinical introduction of new TKR designs and fixation techniques (e.g., new bone cements). The combination of this early and continuous migration defines a specific migration pattern for a specific implant (Pijls and Nelissen 2016). With an increasing number of studies evaluating early migration as well as long-term migration it has now become possible to evaluate the migration pattern between different TKR designs and modes of fixation. This is important since a particular migration pattern may be normal for one TKR design or fixation, but pathological for another TKR design or fixation. It also allows for comparison of the migration measured with different RSA techniques: model-based RSA versus different types of marker-based RSA. The purpose of this systematic review and meta-analysis is therefore to evaluate the early and long-term migration patterns of tibial components of TKR of all known RSA studies.

## Material and methods

This systematic review is reported in accordance with the PRISMA statement (Liberati et al. 2009).

### Literature search

We performed a thorough literature search together with a medical librarian (JP) to reduce bias by increasing the likelihood of retrieving all relevant studies (Vochteloo et al. 2010). The following bibliographies were searched up to July 2016: Pubmed, Embase, Web-of-Science, and the Cochrane Library. Articles in English, French, Italian, Spanish, Dutch, and German were considered. The search strategy consisted of the following components, each defined by a combination of controlled vocabulary and free text terms: (1) RSA, and (2) total knee joint replacement. This search strategy has been used in previously published meta-analyses (Pijls et al. 2012a, 2012b, van der Voort et al. 2015).

### Inclusion and exclusion analysis

Initial screening on the basis of title and abstract of RSA studies was performed by BP to identify studies on patients treated with primary TKRs. When the information in the abstract did not suffice or where there was any doubt, the studies remained eligible. The full text of eligible studies was evaluated by BP with RN as consultant when needed. The inclusion criteria were: (1) primary TKR, and (2) MTPM. MTPM is the unit of measurement for the largest 3D migration of any point on the prosthesis surface (Ryd et al. 1995). Migration pattern was defined as at least 2 postoperative follow-up moments within the first 2 years of follow-up.

Non-clinical studies (animal, phantom) were excluded.

### Data extraction

BP extracted data from the included studies. Mean MTPM with corresponding standard deviation (SD) was extracted or calculated from reported median, inter-quartile range (IQR), or range using internationally accepted methodology (Hozo et al. 2005). Sometimes these values had to be estimated from graphs. In the very rare case that only the mean MTPM was given without range, IQR, SE, or SD, the SD was calculated as the average SD from similar studies. The rationale for this is that it is more important to include data that can later be subjected to sensitivity analyses, rather than to exclude data, which could lead to bias. MTPM at 6 weeks, 3 months, 6 months, 1 year, 2 years, 5 years, and 10 years were considered. Data concerning patient demographics, RSA technique, and prosthesis characteristics (i.e., type of prosthesis, fixation, and insert) were extracted to allow for sub-group analyses.

### Data synthesis and analysis

Prostheses were classified according to prosthesis, fixation and insert (PFI) methodology, as previously used (Pijls et al. 2012a). A study group was defined as a group of patients in a study with the same PFI. Typically these represent the treatment groups of a trial: e.g., a trial comparing fixed versus mobile bearing has 2 study groups. Up to 2-year follow-up we determined and plotted the 10th, 25th, 50th, 75th, and 90th percentile of the means (there were not enough study groups with follow-up beyond 2 years to reliably determine the percentiles).

A random effects model was employed to pool the MTPM of individual study groups in order to estimate an overall MTPM for each follow-up and its associated 95% confidence interval (CI). Random effects meta-regression on study level covariates such as component fixation or mobile versus fixed bearing was employed. MTPM 1 year was used for this meta-regression since it was the most reported value. All analyses were performed using Metafor Package R statistics (Viechtbauer 2010).

We assessed the potential effect of publication bias by comparing the results from the meta-analysis on migration with the results from national joint registries and meta-analyses on revision rates in the discussion.

## Results

The literature search yielded 1,167 hits of which 214 studies remained eligible (953 studies did not study primary TKR). After more detailed evaluation of these eligible studies 10 were excluded because they did not comprise primary TKR, 117 were excluded because they did not mention migration patterns, and 34 were excluded because they were doubles. This left 53 studies to be included, comprising 111 study groups and 2,470 knees (Adalberth et al. 1999, 2000, 2001, 2002, Albrektsson et al. 1990, 1992, Carlsson et al. 2005, Catani et al. 2004, Dalen and Nilsson 2005, Ejaz et al. 2015, Fukuoka et al. 2000, Hansson et al. 2005, 2008, 2009, Henricson et al. 2006, Henricson and Nilsson 2016, Hilding and Aspenberg 2006, 2007, Hilding et al. 1995, Hyldahl et al. 2005, Kienapfel et al. 2004, Ledin et al. 2012, Meunier et al. 2009, Molt et al. 2014, 2016, Molt and Toksvig-Larsen 2014a, 2014b, 2015, Nielsen et al. 1995, Nieuwenhuijse et al. 2013, Nilsson and Dalen 1998, Nilsson and Karrholm 1993, Nilsson et al. 1991, 1999; Petersen et al. 1999, Pijls et al. 2012c, 2012d, Regnér et al. 1998, 2000, Ryd et al. 1986, 1987, 1988, 1993, 1999, Saari et al. 2006, Schotanus et al. 2016, Teeter et al. 2016, Toksvig-Larsen et al. 1994, 1998, 2000, Uvehammer et al. 2007, von Schewelov et al. 2009, Wilson et al. 2012). TKR MTPM at 1 year was the most frequently reported follow-up time, which was reported by all studies. See Table 1 for a breakdown of study groups and knees for each follow-up moment. The median percentage of female patients in each study group was 65% (36–100%). The median percentage of patients with primary osteoarthritis (OA) was 100% (0–100%). 90 study groups were restricted to primary OA. The mean age of study groups varied between 54 years and 76 years with a median of 70 years.

**Table 1.** Number of study groups and knees for each follow-up moment

Follow-up:	Baseline	6 weeks	3 months	6 months	1 year	2 years	5 years	10 years
Study groups	111	53	80	70	111	105	27	8
Knees	2,470	932	1,587	1,318	2,210	2,070	520	86

### Double examinations

Table 2 shows the precision as determined by double examinations from the included studies. 19 of 53 included studies reported original double examinations. The studies that did not report original double examinations often referred to previous studies that reported double examinations or they used phantom experiments.

**Table 2.** Precision as determined by double examinations from the included studies of the meta-analyses

Factor	Studies, n	Mean	Low	High
X translation (mm)	11	0.14	0.06	0.3
Y translation (mm)	19	0.13	0.03	0.22
Z translation (mm)	11	0.20	0.10	0.4
X rotation (°)	18	0.24	0.06	0.6
Y rotation (°)	18	0.34	0.11	0.8
Z rotation (°)	18	0.19	0.06	0.6

MTPM was reported in only 4 studies with the following values:

0.1 mm, 0.2 mm, 0.2 mm, and 0.45 mm.

### Migration results

**Figure 1.**
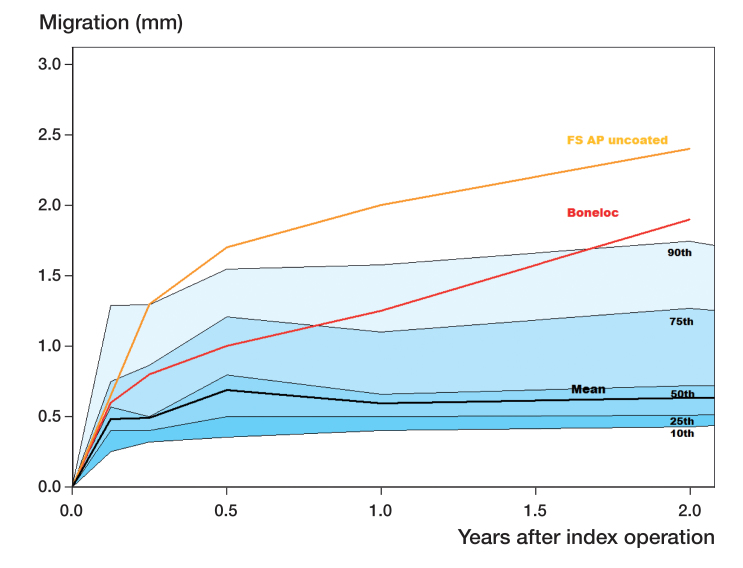
Early migration in percentiles of 111 study groups, 2,470 knees. The migration of two known disasters is also plotted: Boneloc cement (MG II prosthesis) and Freeman–Samuelson all-poly uncoated and uncemented.

**Figure 2.**
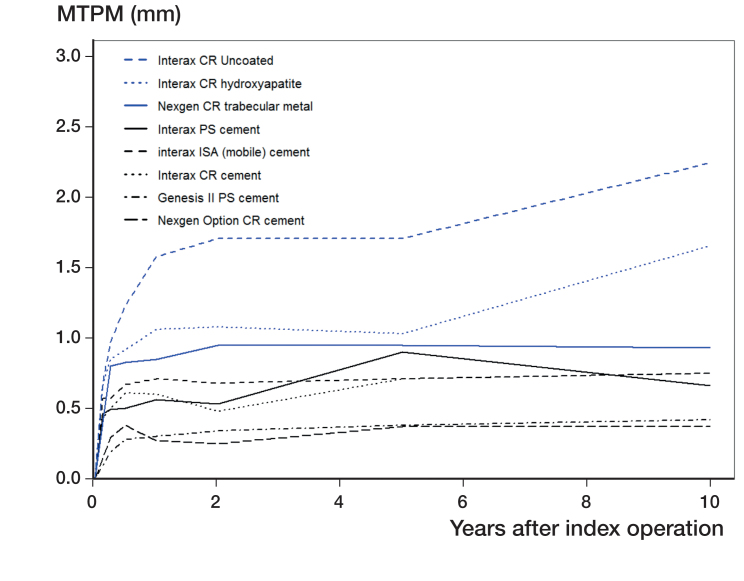
Long-term migration of 8 study groups. Black for cemented TKR. Blue for cementless TKR. CR = cruciate retaining. PS = posterior stabilized.

Early migration in percentiles of the 111 study groups is depicted in Figure 1. The pooled increase in migration between 6 months and 1 year in MTPM is 0.04 mm (CI 0.02–0.07) based on 70 study groups. The pooled increase in MTPM migration between 1 year and 2 years is 0.04 mm (CI 0.02–0.06) based on 105 study groups (6 studies did not report 2-year data). To validate our assumption, the MTPM migration pattern throughout follow-up of 2 known orthopedic implant disasters is also plotted in Figure 1: Boneloc cement (MG II TKR prosthesis) and Freeman–Samuelson uncoated and uncemented TKR. 8 study groups reported MTPM migration results up to 10 years’ follow-up (Figure 2). In these long-term studies, the majority of TKR stabilized during follow-up, although 2 uncemented types of TKR continued to migrate.

**Figure 3.**
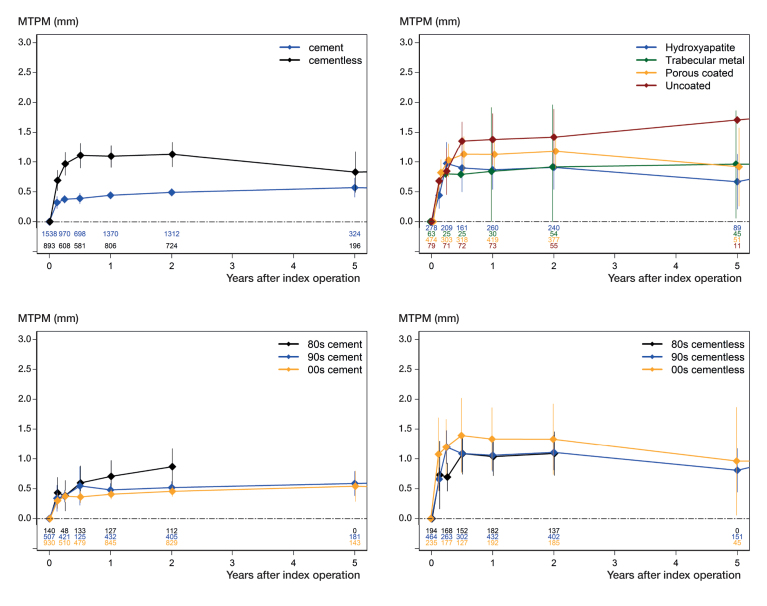
Migration patterns for cemented and cementless TKR, types of cementless TKR and migration according to decade in which the inclusion of the study started for cemented and cementless TKR separately. The number of RSA examinations is given for each follow-up in color and order corresponding to the legend.

The pooled MTPM 1 year of cemented TKR was less than that of uncemented TKR: 0.44 mm (CI 0.38–0.50) compared with 1.09 mm (CI 0.91–1.28) (Figure 3). The migration patterns of different fixation types of uncemented TKR are shown in Figure 3.

**Figure 4.**
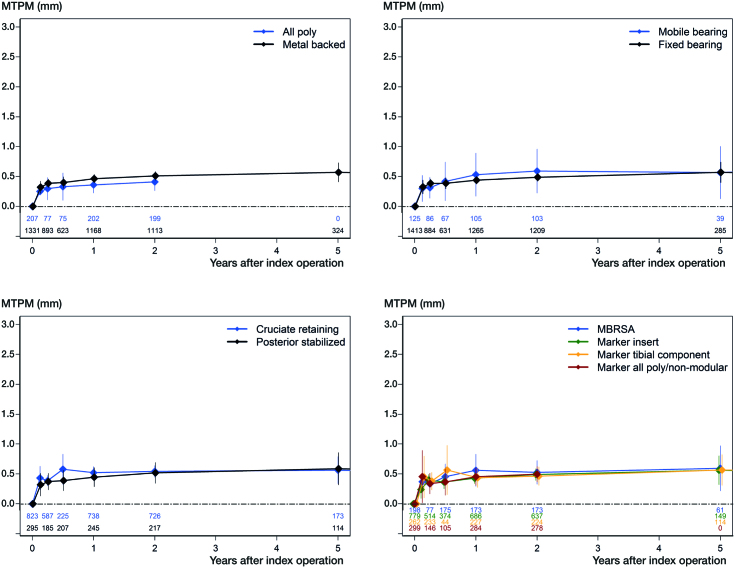
Migration patterns for cemented TKR. Comparisons of all poly versus metal backed, mobile bearing versus fixed bearing, cruciate retaining versus posterior stabilized and RSA techniques is made. The number of RSA examinations is given for each follow-up in color and order corresponding to the legend. MBRSA = model-based RSA. For a detailed description on RSA techniques see the text.

There was some variation for MTPM 1 year with the most migration for uncoated TKR, but the confidence intervals overlapped: hydroxyapatite coated 0.87 mm (CI 0.54–1.20), trabecular metal 0.84 mm (CI 0–1.92), porous coated 1.13 mm (CI 0.87–1.38), and uncoated 1.38 mm (CI 0.95–1.82). Comparing TKR migration data throughout the last 35 years, for cemented TKR migration decreased during the last decades, with MTPM at 1 year in the 1980s being 0.70 mm (CI 0.44–0.97), in the 1990s 0.48 mm (CI 0.37–0.59), and in the 2000s 0.41 mm (CI 0.33–0.49) (Figure 3). For the uncemented TKR the migration increased during these 35 years, MTPM at 1 year in the 1980s being 1.04 mm (CI 0.72–1.35), in the 1990s 1.06 mm (CI 0.79–1.33), and in the 2000s 1.33 mm (CI 0.81–1.86) (Figure 3). There seemed to be no difference in mean migration between cemented all-poly and cemented metal-backed TKR with MTPM 1 year 0.36 mm (CI 0.22–0.49) and 0.46 mm (CI 0.39–0.53) (Figure 4). There seemed to be no difference in migration between cemented mobile bearing and cemented fixed bearing TKR, MTPM 1 year being 0.53 mm (CI 0.17–0.89) and 0.44 mm (CI 0.37–0.50) (Figure 4). There seemed to be no difference in migration between cemented cruciate retaining and cemented posterior stabilized TKR with MTPM 1 year 0.52 mm (CI 0.42–0.62) and 0.45 mm (CI 0.29–0.60) (Figure 4). For cemented TKR there seemed to be no difference in migration between RSA techniques used. Pooled MTPM 1 year was 0.56 mm (CI 0.29–0.83) for model-based RSA (MBRSA), 0.43 mm (CI 0.34–0.51) for marker-based RSA with markers in the modular polyethylene (PE) insert, 0.43 mm (CI 0.27–0.60) for marker-based RSA with markers attached to the tibial baseplate or tibial stem and 0.45 mm (CI 0.31–0.60) for model-based RSA with markers in the PE of all-poly TKR or markers in the PE of non-modular TKR (Figure 4).

## Discussion

The results from this systematic review and meta-analysis of 2,470 TKRs show that the majority of early migration occurs in the first 6 postoperative months followed by a period of no or very little migration within the bone (i.e., plateau phase). From 6 months to 1 year there was on average 0.04 mm migration, which was similar to the migration between 1 year and 2 years of 0.04 mm.

**Figure 5.**
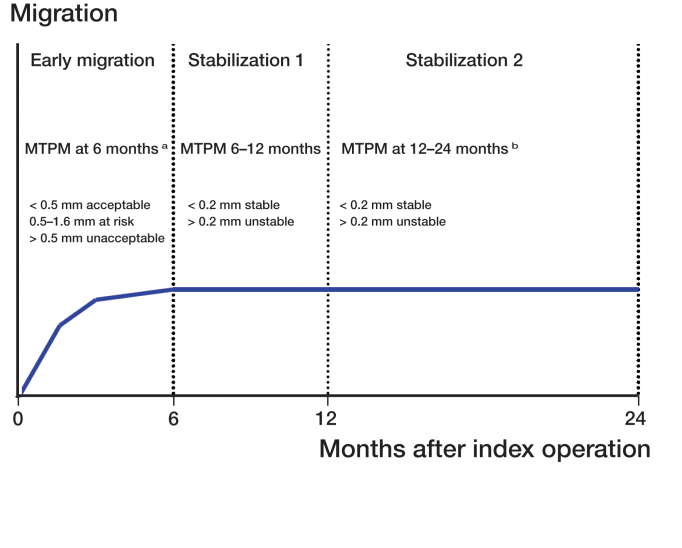
Proposed RSA migration evaluation for 2 years’ follow-up based on the results of the meta-analyses. Early migration is evaluated by MTPM at 6 months, stabilization 1 is evaluated by MTPM between 6 and 12 months, and stabilization 2 is evaluated by MTPM between 12 and 24 months. **^a^** modified according to Pijls et al. 2012a. **^b^** according to Ryd et al. 1995.

Given this similarity between MTPM 6 months and MTPM 1 year, we propose using the MTPM 6 months values (instead of MTPM 1 year values) for RSA threshold testing (Pijls et al. 2012a) (Figure 5). Since the TKR migrated only 0.04 mm between 6 months and 1 year, the MTPM 6 months is almost identical to the MTPM 1 year (MTPM 6 months = MTPM 1 year—0.04 mm), which is currently used as a threshold for RSA studies (Pijls et al. 2012a). Therefore, the MTPM 6 months values can be subjected to the MTPM 1 year thresholds of less than 0.5 mm for acceptable migration and more than 1.6 mm for unacceptable migration.

The migration between 6 months and 12 months can then be used to evaluate the presence of migration stabilization for the “at risk” and “unacceptable” groups, using the 0.2 mm values of Ryd et al. (1995). Nevertheless, the migration between 1 and 2 years can be used as an extra safeguard during the phased introduction of TKR implants. It is also important to have migration results before 6 months (e.g., 6 weeks and 3 months) to evaluate the migration pattern.

Cemented TKR have very little early migration (MTPM 1 year 0.44 mm) most likely because they rely on primary bone fixation through cement interdigitation. The migration of Boneloc cement, a historical disaster, is very high with MTPM 1 year 1.25 mm compared with the mean 0.44 mm for all cemented TKR. Furthermore, TKR with Boneloc did not stabilize between 1 and 2 years. In retrospect, with the data from this meta-analysis it would already have been possible to identify Boneloc bone cement as a potential disaster at 6 months’ RSA follow-up and using sequential RSA migration follow-up data to evaluate whether a migration stabilization phase occurs.

For cemented TKR there seemed to be no difference in migration between all-poly and metal-backed TKR for the first 6 postoperative months or for the period beyond this time point up to 2 years postoperatively. These data on the low migration of the all-poly tibial component are in line with Nouta et al. (2012) who found no difference in revision for aseptic loosening between all-poly and metal-backed TKR at long-term follow-up. For cemented TKR there seemed to be no difference in migration between mobile-bearing TKR and fixed-bearing TKR up to 5 years’ follow-up. These results are in line with a recent Cochrane review showing no differences in revision rates between fixed- and mobile-bearing TKR (Hofstede et al. 2015). Apparently, the theoretical advantage of mobile-bearing TKR in reducing the stress on the bone–cement–prosthesis interface does not translate into less migration of the tibial component or fewer revisions for aseptic loosening of cemented TKR. For cemented TKR there seemed to be no difference in migration between cruciate retaining and posterior stabilized TKR up to 5 years’ follow-up. These results are in line with a recent Cochrane review showing no differences in revision rates between cruciate retaining and posterior stabilized TKR (Verra et al. 2013).

As for migration measurements of orthopedic implants within bone, 2 RSA measurement techniques exist, a manual, marker-based RSA method and a semi-automatic CAD model-based RSA (MBRSA) technique (Valstar et al. 2000, Hurschler et al. 2009). There have been concerns that model-based RSA (MBRSA) might overestimate migration results compared with marker-based methods for the same analysis because MBRSA uses CAD models with thousands of points (triangles) compared with 3–5 tantalum markers for marker-based methods (Tjornild et al. 2015). The marker or point within a segment that has moved the most provides the MTPM. Our results show that, for the included TKR, there seemed to be no difference in migration measured with MBRSA or 3 types of marker-based methods (e.g., markers in tibial insert, attached to the tibial base plate). Between the 3 marker-based RSA methods there was no difference in migration for cemented TKR. We evaluated marker-based RSA that uses markers in the PE insert of modular TKR, marker-based RSA that uses markers attached to the tibial baseplate, and marker-based RSA that uses markers in the PE insert of non-modular TKR or all-poly TKR. This is particularly important for marker-based RSA using markers in modular tibial inserts, since movement between the insert and tibial baseplate could appear as migration of the TKR relative to the bone when this is actually migration of the insert within the tibial base plate and thus backside wear and creep of the poly insert. This could theoretically give higher migration values for TKR with markers in modular inserts, whilst it is actually backside wear. Our results show that up to 5 years’ migration results, this effect is negligible and thus migration results are actually the migration of the tibial component within the bone.

Uncemented TKR have high early migration (MTPM 1 year 1.09 mm) followed by stabilization of this migration. The latter is most likely due to the secondary fixation by bone in/ongrowth of these uncemented components. The migration of the uncemented uncoated FS AP, a historical disaster with early loosening, is very high and does not stabilize at all during follow-up, either at the 6 months’ mark or at the 2-year mark. It had one of the worst ever revision rates recorded in national registries (Knutson et al. 1986). In retrospect with the data from the current and previous meta-analysis it would already have been possible to identify the uncemented FS as a potential disaster at 6 months’ RSA follow-up using sequential RSA follow-up to evaluate whether stabilization of this progressive migration occurs. For uncemented TKR there was some variation in migration with the highest migration for the uncoated TKR, although the 95% confidence intervals overlapped. During the last 35 years the migration of uncemented TKR seemed to increase. A possible explanation for this phenomenon is the fact that 5 out of 8 PFIs from the 2000s are porous coated.

Regarding precision of the RSA measurements, only 19 of 53 included studies reported precision as determined by original double examinations. It is important to know the precision of RSA measurements. Therefore future RSA studies should perform double examinations as is also required by the RSA guidelines (Valstar et al. 2005).

A limitation of this meta-analysis may be that for the direct comparisons of different types of TKR (e.g., mobile versus fixed bearing) pooled results of different trials might have been more appropriate. However, continuous migration data or absence of a migration stabilization phase as shown in this meta-analysis are in line with the revision rates for these TKR types from meta-analyses and the survival analysis results of national joint registries. We focused on MTPM of the TKR, although translations and rotations migration measurements could have been of interest as shown by Gudnason et al. (2017). However, since translations and rotations were not reported in a uniform manner, a meaningful analysis is not possible. Future studies could benefit from further standardization regarding reporting of the results. The disadvantage of MTPM is that it gives information only on the magnitude of the migration, not on the direction of the migration. The latter could also be of interest and may even be better than MTPM (Gudnason et al. 2017). Nevertheless, MTPM does have advantages. It evaluates the fundamental assumption of loosening: is the prosthesis loose or not? Also it is the most reported parameter, going back to the beginning of RSA, and the present meta-analysis includes 2,470 knees with MTPM.

In summary the results from this meta-analysis on RSA migration are in line with the results of national implant registries as well as the results of meta-analyses on revision rates, providing further proof for the association between early implant migration and late revision for aseptic loosening of TKR. The pooled migration patterns can be used both as benchmarks as well as for defining migration thresholds for future evaluation of new TKR and fixations. Thus RSA has a place for a safe premarket approval evaluation and as such should be part of a phased introduction of new implants (Nelissen et al. 2011). With the data from this meta-analysis it appears possible (Figure 5) to have a first evaluation of the safety (i.e. implant–bone fixation) of the implant at 6 months.

BGP and RGN conceived the study. JWP designed the search strategy for the literature search. BGP performed the study selection, data extraction, and analyses. RGN ensure accuracy of data extraction, was the referee and helped with interpretation of the results. BGP, JWP, and RGN wrote the manuscript.

Data extraction of the RSA studies and results from the meta-analyses are available by contacting the corresponding author.

No conflicts of interest declared.

No external funding.

*Acta* thanks Mogens Berg Laursen and Stephan Röhrl for help with peer review of this study.
